# Bioactive Properties of *Tagetes erecta* Edible Flowers: Polyphenol and Antioxidant Characterization and Therapeutic Activity against Ovarian Tumoral Cells and *Caenorhabditis elegans* Tauopathy

**DOI:** 10.3390/ijms25010280

**Published:** 2023-12-24

**Authors:** Lorenzo Rivas-García, Lara Crespo-Antolín, Tamara Y. Forbes-Hernández, Jose M. Romero-Márquez, María D. Navarro-Hortal, Miguel Arredondo, Juan Llopis, José L. Quiles, Cristina Sánchez-González

**Affiliations:** 1 Department of Physiology, Institute of Nutrition and Food Technology “José Mataix Verdú”, Biomedical Research Centre, University of Granada, 18016 Armilla, Spain; lorenrivas@ugr.es (L.R.-G.); lcall95@gmail.com (L.C.-A.); romeromarquez@ugr.es (J.M.R.-M.); mdnavarro@ugr.es (M.D.N.-H.); jllopis@ugr.es (J.L.); jlquiles@ugr.es (J.L.Q.); 2 Sport and Health Research Centre, University of Granada, 18016 Armilla, Spain; 3 Micronutrient Laboratory, Institute of Nutrition and Food Technology, University of Chile, Santiago 7830490, Chile; miguel.arredondo@inta.uchile.cl; 4 Research Group on Foods, Nutritional Biochemistry and Health, Universidad Europea del Atlántico, 39011 Santander, Spain

**Keywords:** cancer, tau protein, mitochondria, polyphenols, apoptosis, oxidative stress

## Abstract

*Tagetes erecta* is an edible flower deeply rooted in traditional Mexican culture. It holds a central role in the most popular and iconic Mexican celebration, “the Day of the Dead”. Furthermore, it is currently receiving interest as a potential therapeutic agent, motivated mainly by its polyphenol content. The present study aims to evaluate the biological activity of an extract synthesized from the petals of the edible flower *T. erecta*. This extract showed significant antioxidant scores measured by the most common in vitro methodologies (FRAP, ABTS, and DPPH), with values of 1475.3 μM trolox/g extr, 1950.3 μM trolox/g extr, and 977.7 μM trolox/g extr, respectively. In addition, up to 36 individual polyphenols were identified by chromatography. Regarding the biomedical aspects of the petal extract, it exhibited antitumoral activity against ovarian carcinoma cells evaluated by the MTS assay, revealing a lower value of IC_50_ compared to other flower extracts. For example, the extract from *T. erecta* reported an IC_50_ value half as low as an extract from *Rosa* × *hybrida* and six times lower than another extract from *Tulbaghia violacea*. This antitumoral effect of *T. erecta* arises from the induction of the apoptotic process; thus, incubating ovarian carcinoma cells with the petal extract increased the rate of apoptotic cells measured by flow cytometry. Moreover, the extract also demonstrated efficacy as a therapeutic agent against tauopathy, a feature of Alzheimer’s disease (AD) in the *Caenorhabditis elegans* experimental model. Treating worms with the experimental extract prevented disfunction in several motility parameters such as wavelength and swimming speed. Furthermore, the *T. erecta* petal extract prevented the release of Reactive Oxygen Species (ROS), which are associated with the progression of AD. Thus, treatment with the extract resulted in an approximate 20% reduction in ROS production. These findings suggest that these petals could serve as a suitable source of polyphenols for biomedical applications.

## 1. Introduction

Edible flowers have received more attention during recent years, motivated not only by their culinary properties which add a unique texture, color, and taste to modern recipes such as soups, salads, and desserts, but also for their high amount of polyphenols which could conditionate their uses in therapy [1]. Thus, consumers seek edible flowers, and their commercialization has increased exponentially. In fact, these new foods have become a “fashion ingredient” in some countries such as the United Kingdom and Australia [2,3]. However, consumers perceive the information on edible flowers to be limited. In this line, Guiné et al. described the perception of this edible flower in a European country (Portugal) and a Latin American country (Costa Rica), and more than 90% of participants stressed that the availability of information about this type of food is insufficient [4]. Thus, it is necessary to contribute to unraveling the effects of edible flowers on human metabolism, in order to synthesize functional food products based on these ingredients.

*Tagetes erecta*, commonly known as the Mexican marigold, is a Mexican plant that is deeply rooted in the popular culture of this North American country. This flower plays a key role in the country’s most iconic celebration, the “Day of the Dead”. In this commemoration, Mexican marigold petals decorate paths and shrines in Mexican homes. Some authors have studied the possible biological activities of extracts obtained from the petals of Mexican marigolds [5]. They reported effects against Alzheimer’s disease-related amyloidosis in worms [6] and described its gastric healing activity in a murine model [7]. These activities could be associated with various chemical components including carotenoids [7] and polyphenols [6]. Some authors have identified lutein (a carotenoid) and laricitrin (a flavonoid) as the main chemical structures abundant in *T. erecta* and responsible for its biological activities [8].

The incidence of aging-associated diseases like cancer and AD has increased exponentially in recent years, especially in Western countries. Several authors have proposed that according to the oxidative theory of aging, some of the age-related damage could be mediated by the accumulation of ROS [9,10,11,12]. The relationship between cancer and ROS is complex and depends on the context. However, some authors have proposed that reducing ROS levels could decrease tumor progression and metastatic activity in different types of cancers [13]. The connection between ROS accumulation and tumorigenesis could be explained in two ways: on one hand, the increment of ROS could directly oxidate cellular macromolecules like proteins, nucleic acids, and/or lipids and promote gene mutation and consequently induce the inflammation process [14]. However, other authors propose that ROS accumulation could activate aberrant signaling cellular pathways associated with tumorigenesis [15]. Furthermore, oxidative stress may be implicated in different neurodegenerative disorders like AD or Parkinson’s disease. Hence, high ROS levels could be found even in the early stages of these diseases [16,17,18]. An unbalanced ratio between antioxidants and pro-oxidants could activate lipid peroxidation and protein damage, altering the metabolism of hippocampal and parietal cortex pyramidal neurons [19].

Edible flowers have been proposed as a source of polyphenols with antioxidant activity, such as kaempferol, quercetin, and myricetin [1]. Their implementation in diet revealed chemo-preventive effects mediated by modulation of apoptosis, reducing the cell cycle progression and mitigating the metastatic tumoral process [20]. Several studies strongly suggest that long-term consumption of polyphenols through diet or functional foods offers protection against the development of various chronic diseases [21]. Thus, edible flowers have recently been postulated as a novel way for functional food development [6,22,23,24]. As previously mentioned, the present work aims to evaluate the potential biological effect of petals from the edible flower *T. erecta*, assessing antioxidant activity and the phenolic profile of an ethanolic extract obtained from these petals. Furthermore, it describes the effects of this extract as therapeutic agents against two age-dependent diseases: cancer and AD.

## 2. Results and Discussion

### 2.1. Petal Extract Characterization

The increasing interest in studying the biological properties of Mexican marigold petals is primarily due to their high polyphenol content, which has been described in multiple species of edible flowers [1]. TPC and TFC were estimated for the Mexican marigold extract, as these secondary metabolites are widespread in plants and their amount could be related to their possible implications in human health. The extract exhibited a phenolic value of 81.1 ± 10.6 mg GAE/g extr (Gallic Acid Equivalent) and a score of total flavonoid content of 23.17 ± 4.26 mg catechin/g extr (Table 1). Thus, the extract showed a TPC score similar to other *T. erecta* extracts [25,26]. However, the value of this extract is higher than that obtained from other edible flowers; in fact, a methanolic extract of *Tulbhagia violacea* [23] and *Rosa* × *hybrida* [22] exhibited a lower amount of TPC, and an ethanolic extract of *Viola cornuta* and *Viola* × *wittrockiana* showed a reduced TPC score [27]. Therefore, *T. erecta* could be considered one of the edible flowers with a greater TPC capacity. Regarding TFC, the score for the *T. erecta* extract was similar to others obtained from edible flowers [22,23].

Furthermore, the antioxidant activity of the Mexican marigold extract was assessed using three different methodologies (FRAP, DPPH, and ABTS), reporting values of 1475.3 ± 122.5 μM trolox/g extr, 1950.3 ± 211.1 μM trolox/g extr, and 977.7 ± 62.4 μM trolox/g extr, respectively. Generally, the biological activity of Mexican marigold petals has been related to their antioxidant capacity [28]. Therefore, the analgesic effect and phytosimulation ability of these petals are attributed to their high antioxidant capacity [29,30]. The extract assayed in the present study reported a similar score for the antioxidant parameters compared with other extracts synthesized from edible flowers. For example, the FRAP, DPPH, and ABTS values for the present extract were comparable to methanolic extracts from *Rosa* × *hybrida*, *Viola cornuta,* or *Tulbaghia violacea* petals [22,23].

**Figure 1:**
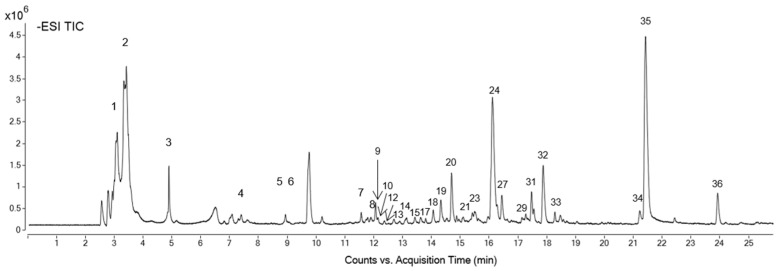
The total ion chromatogram of *T. erecta* extract at a concentration of 1 mg/mL. Numeration of chromatographic peaks is according to Table 2.

Subsequently, the analysis and identification of the compounds of the extract were carried out by HPLC-ESI-QTOF-MS/MS at a concentration of 1 mg/mL. The total ion chromatogram (TIC) obtained by analyzing the extract is shown in Figure 1. Thirty-six isolated chemical compounds were identified and quantified (Table 2). Deoxy-fructofuranosyl deoxy-glucopyranoside/fructofuranosyl dideoxy-xylo-hexopyranoside, patulitrin, methoxyquercetin, and isorhamnetin hexoside exhibited concentrations higher than 20 mg/mL.

It is noteworthy that the effectiveness of polyphenol extraction from *T. erecta* petals can be significantly influenced by the choice of solvents and their polarity. Polyphenols exhibit varying degrees of polarity, and selecting solvents with appropriate polarity is crucial for efficient extraction [31]. Generally, polar solvents such as ethanol or methanol are often effective in extracting a broad range of polyphenols due to their ability to interact with polar functional groups. For instance, Burlec identified up to 57 individual compounds in a methanolic extract of Mexican marigolds [28] using methanol as a solvent, whereas when the extraction solvent was formed from a mixture of ethanol and water in different proportions, the number of individual polyphenols identified reduced. The results we obtained partially agreed with those reported by other authors. Some compounds including laricitrin, isorhamnetin, and ellagic acid have already been found in other extracts obtained from the *T. erecta* plant [25,26,32]. However, their distribution differed partially from what we obtained for the present extract. For example, some authors identified myricetin and sinapic acid as two of the main components of the petal extracts and proposed ellagic gallic acid as a minority component [26], and other authors corroborated this hypothesis [6]. However, under the present conditions, myricetin and gallic acid were not identified. This discrepancy could be attributed to the dissolvent used in the extract synthesis procedure. Conversely, the majority of studies concluded that lacritirin is the predominant component in *T. erecta* petals [25], and this is also the major constituent in the extract of the present study (Table 2). Therefore, the reported biomedical properties of this flower might be primarily attributed to this polyphenol.

### 2.2. In Vitro Assays

**Figure 2:**
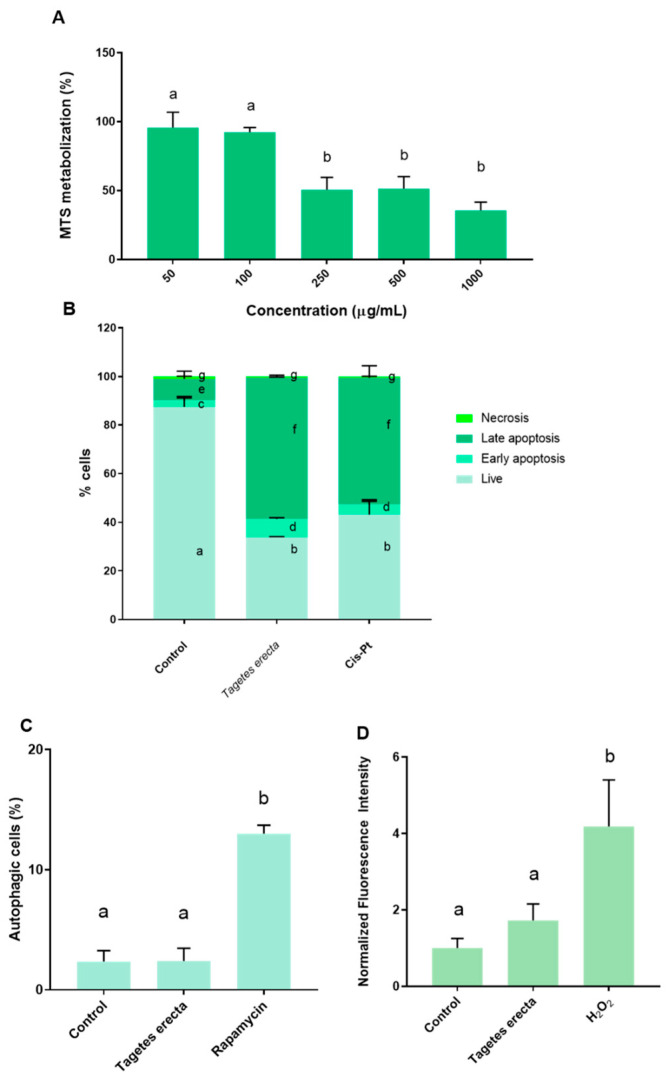
Effect of 48 h exposition of ethanolic *T. erecta* extract. (**A**) Viability assay (MTS); (**B**) Apoptosis/necrosis measured by flow cytometry. IC_50_ of cis-Pt used as positive control; (**C**) ROS production. H_2_O_2_ 25 µM used as positive control; (**D**) Autophagy induction. Rapamycin 0.5 µmol L^−1^ as positive control. Results are expressed as mean ± SD. For each parameter, different letters indicate statistically significant differences between groups (*p* < 0.05).

Firstly, the antitumor capacity of the extract obtained from the *T. erecta* petals was assessed with the MTS assay, which is widely used to evaluate antitumor compounds. Ovarian tumor cells were incubated with increasing concentrations of the extract for 48 h. In this case, lower concentrations (50 μg/mL and 100 μg/mL) did not induce significant changes in cellular viability compared to the control. However, a dose-response effect was observed starting from the 250 μg/mL concentration; treatment with the 250 μg/mL resulted in a lower cellular viability of approximately 50%. A similar effect was observed with the dose of 500 μg/mL. For the highest dose (1000 μg/mL), viability was approximately 60% lower. The doses of 250 μg/mL, 500 μg/mL, and 1000 μg/mL led to statistically significantly lower cellular viability compared to both the control and the lower doses (50 μg/mL and 100 μg/mL) (Figure 2A). Subsequently, the IC_50_ of the extract was 144.5 µg/mL.

Ovarian cancer is the cancer with the fourth highest incidence rate in women and is related to high mortality mediated by late diagnosis and drug resistance of conventional drug therapy [33]. To overcome these problems, polyphenols obtained from plants have recently been proposed as an alternative tool for antitumoral drug development [34]. The antitumoral activity of the Mexican marigold extract was tested by the MTS assay. Figure 2A shows the effect of the petal extract on ovarian tumoral cell viability. In this case, doses between 250–1000 µg/mL reported antiproliferative effectivity in a dose-dependent manner. The most effective dose was 1000 µg/mL, which reduced the tumoral cell viability to below 50%. The IC_50_ of the extract was 144.5 µg/mL. The antitumoral activity of *T. erecta* has previously been elucidated against other types of tumors such as breast cancer cells [35], hepatocarcinoma [36], lung cells [37], colorectal tumoral cells [37], gastric cancer [38], and prostatic tumoral cells [39]. However, the present work first reported the antitumoral activity of *T. erecta* against ovarian tumoral cells. The value of IC_50_ obtained is in agreement with the other authors described (normally IC_50_ is between 150–400 µg/mL, depending on the cancer cell line and the part of the plant used). Moreover, this extract reported an IC_50_ against ovarian tumoral cells lower than others obtained from edible flowers. Thus, the *T. erecta* extract had an IC_50_ eight times lower than *Tulbaghia violacea* [23] and half that of *Rosa* × *hybrida* [22]. These results suggested that the antitumoral activity (IC_50_) could be inversely related to the total flavonoid content; in this way, the total flavonoid amount of the extract (Table 1) was approximately ten times higher than the *Tulbaghia violacea* extract [23]. In order to obtain a better understanding, the cell death mechanisms were evaluated. A2780 cells were incubated with the IC_50_ of the petal extract for 48 h. This treatment resulted in a significantly higher percentage of apoptotic cells (late and early apoptosis stages). Figure 2B shows that cells treated with the petal extract had statistically significantly less live cells than untreated cells. Moreover, treatment with the extract led to a significantly higher ratio of apoptotic cells (early and late) compared with the untreated cells (Figure 2B). Furthermore, treating ovarian tumoral cells with the extract resulted in a cell distribution similar to that produced by the positive control (IC_50_ of cis-Pt), one of the drugs usually used in ovarian cancer therapy. The induction of apoptosis by extracts obtained from *T. erecta* has previously been described by other authors. Gansukh et al. evidenced that the lutein extracted from *T. erecta* activated apoptosis against human cervical carcinoma, increasing the activity of caspase-3, downregulating the Bcl-2 (antiapoptotic), and upregulating BAX (proapoptotic) [40]. The proapoptotic activity obtained in the present conditions could be associated with the content of the extract in ellagic acid. Thus, some authors have described the role of ellagic acid in the induction of apoptosis via the inhibition of Cyclin-Dependent Kinase 6 [41], an enzyme that plays a key role in cancer progression and could be used as a drug target in anticancer therapeutics [42]. Thus, developing a new pharmaceutical platform for controlling extract delivery could be a new tool for oncological therapy. In contrast, incubation with the extract did not cause significant changes in the induction of autophagy between treated cells and control cells (Figure 2C), and incubation with the *T. erecta* extract did not lead to significant changes in ROS production (Figure 2D). These results are in disagreement with those observed in other extracts obtained from edible flowers. Thus, *Tulbhagia violacea* and *Rosa* × *hybrida* extracts reported antiproliferative activity against ovarian tumoral cells by the induction of autophagy and ROS [22,23].

### 2.3. Caenorhabditis elegans Assays

In recent years, the potential impact of certain subproducts/waste products obtained from the food industry on neurodegenerative diseases has grown [43,44], mainly due to their high polyphenol content.

*C. elegans* model was employed with dual objectives: ensuring the absence of toxicity of the extract and evaluating its potential application for AD. Previous studies suggested the lack of toxicity of *T. erecta* extracts. C. Moliner and colleagues demonstrated that increasing doses of up to 2000 mg/mL of a methanolic extract synthesized from *T. erecta* petals provided by the same supplier of the present study did not alter the viability of wild-type worms [6]. Additionally, they showed that *T. erecta* extract did not modify the lifespan of worms [6]. Furthermore, Hamaguchi et al. confirmed not only the lack of toxicity of an allelochemical obtained from *T. erecta* but also its ability to ameliorate oxidative stress [45]. Similar results were reported by other authors [46]. To complete the toxicological knowledge of *T. erecta*, the acute lethality in wild-type worms and some locomotive parameters of the tauopathy nematodes were measured.

**Figure 3:**
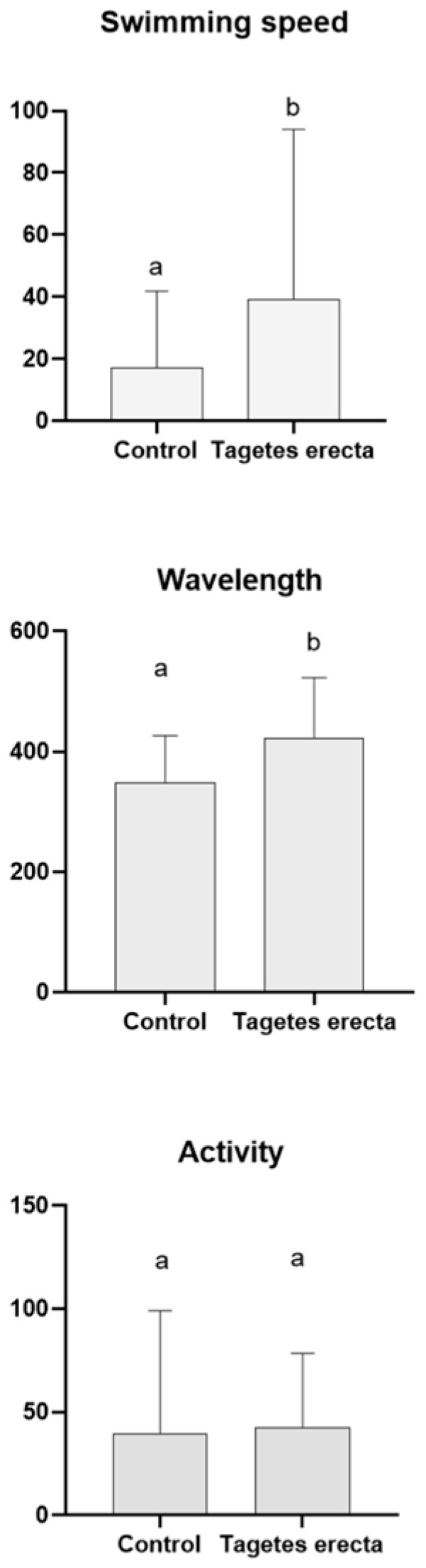
Motility parameters of worms. Mean ± SD. For each parameter, different letters indicate statistically significant differences between groups (*p* < 0.05).

Supplementary Figure S1 shows that all the doses of *T. erecta* employed were not lethal for the wild-type worms. These results agreed with those previously proposed [6]. A non-lethal submaximal concentration (100 μg/mL) of Mexican marigold extract was then employed for the tests. Subsequently, other parameters were evaluated in a tauopathy worm model. In this case, treatment with the Mexican marigold extract for 72 h resulted in a statistically significant improvement in the swimming speed and wavelength parameters compared to untreated worms (Figure 3). Thus, treating the nematodes with the flower extract led to faster swimming speed and higher wavelength. However, no changes were observed in the activity parameter between the two groups (treated and untreated).

These findings suggest that *T. erecta* not only exhibited no toxicity towards the worms but also enhanced certain measures of mobility. Consequently, these flowers reported anti-neurodegenerative activity, in agreement with previous studies which proposed the absence of Mexican marigold toxicity using a *C. elegans* model [6]. For example, several authors have previously described how the polyphenols present in *T. erecta* could mitigate the damage induced by juglone, a potent oxidative agent [6]. They reported that increasing doses of Mexican marigold extract protected against the cumulative stress caused by juglone in N2 wild-type *C. elegans* [6]. Furthermore, a polyphenol isolated from Mexican marigold petals prevented ROS generation caused by treatment with a hepatotoxin (mycrocystin-LR) [46]. In that case, including the lutein obtained from Mexican marigold petals corrected the levels of oxidative stress markers produced by microcystic-LR in a *C. elegans* model [46].

Furthermore, the effect of Mexican marigold petal extract on AD was studied using a worm model. In this case, the BR5706 strain was used, which exhibits an alteration in the phosphorylation and aggregation of the human tau protein in neurons [47]. The tau protein plays a crucial role in AD and is closely associated with the neuropathological characteristics of the disease [48]. Under normal conditions, tau protein stabilizes and provides support to microtubules in nerve cells. However, in AD, the tau protein undergoes modifications and accumulates in the form of neurofibrillary tangles inside brain cells [48]. These tangles disrupt the normal function of cells and eventually contribute to their death. Additionally, these tangles can interfere with communication between brain cells, leading to the cognitive dysfunction associated with AD [49]. Therefore, understanding the roles of tau protein and its involvement in AD will enable targeted treatments mitigating its harmful effects to be developed. Other authors have previously demonstrated the potential therapeutic applications of Mexican marigold petals in treating AD. Consequently, they demonstrated that treating the CL4176 *C. elegans* strain (which could induce the accumulation of β-amyloid peptide) resulted in a delay in the paralysis of these worms [6]. However, the impact of Mexican marigold petals on tau protein has not previously been documented. Our results clearly demonstrated that treating tauopathy-afflicted worms with Mexican marigold extract led to significant improvements in various indicators of locomotion, including swimming speed and wavelength (Figure 3). Recent research suggests that polyphenols in food can effectively mitigate the detrimental effects of AD in a tauopathy model. In this way, some extracts obtained from the food industry have proven their efficacy in this worm model. Extracts derived from honey displayed the potential to enhance mobility parameters in BR5706 worms [50,51]. For example, an avocado honey extract increased stretching efforts, swimming speed, and overall activity in BR5706-treated worms compared to untreated ones [51]. Furthermore, subproducts sourced from *Olea europaea* also exhibited beneficial effects on these types of parameters [52]. To date, our findings represent the first report of the potential therapeutic activity of a *T. erecta* extract in a tauopathy model.

**Figure 4:**
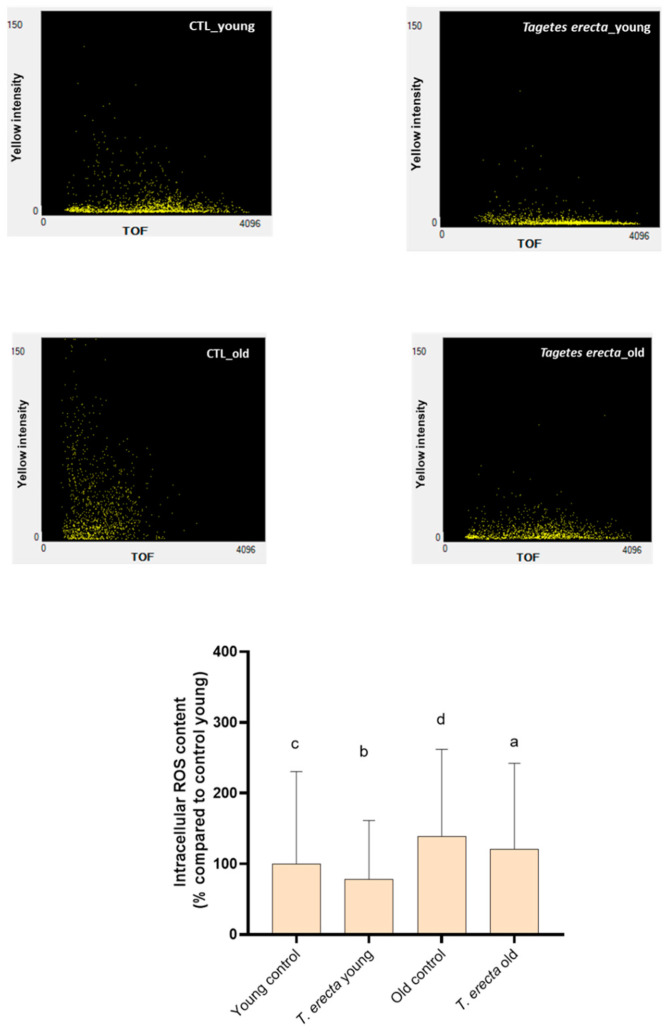
Intracellular ROS content in Young and Old worms. Intracellular ROS content was measured by the 2′,7′-dichlorofluorescein diacetate (H2DCFDA) assay. L3 stage synchronized N2 nematodes were treated or untreated with 100 µg/mL *T. erecta* extract and incubated at 20 °C until the read-out point of the experiment (Young worms: 5 days old; Old worms: 12 days old). Representative images of Cell ROS quantification by Multi-Range Large Particle Flow Cytometer Biosorter (Union Biometrica, MA, USA) are shown above the bar charts (yellow fluorescence intensity vs. TOF: time of flight; worm size). Data are expressed as mean ± SD of three independent experiments (n = 3). Lower-case letters, when different, indicate statistically significant differences (*p* < 0.05). CTL_young: Young control (untreated worms); *T. erecta*_young: *T. erecta* treated worms (Young); CTL_old: Old control (untreated worms); *T. erecta*_old: *T. erecta* treated worms (Old).

AD and cancer are two of the most prominent diseases associated with aging [10,53]. Aging is a natural and inevitable process that affects all living organisms, during which cells and tissues undergo a series of changes that make them more susceptible to a wide range of diseases [10]. One of the key factors contributing to the disease development in the context of aging is oxidative stress [9]. The effect of the Mexican marigold extract on the liberation of ROS was evaluated through fluorescence. This measurement was performed under two physiological conditions: aging, represented by 12-day-old worms, and youth, represented by 5-day-old worms. In both cases, the inclusion of the extract partially prevented ROS production compared to untreated worms (Figure 4). Thus, the extract obtained from the Mexican marigold petals significantly prevented the generation of ROS, indicating that the protective effect exerted by this extract may be associated with its antioxidant capacity. Previous studies have also indicated that Mexican marigold petals have the ability to mitigate oxidative damage [46].

**Figure 5:**
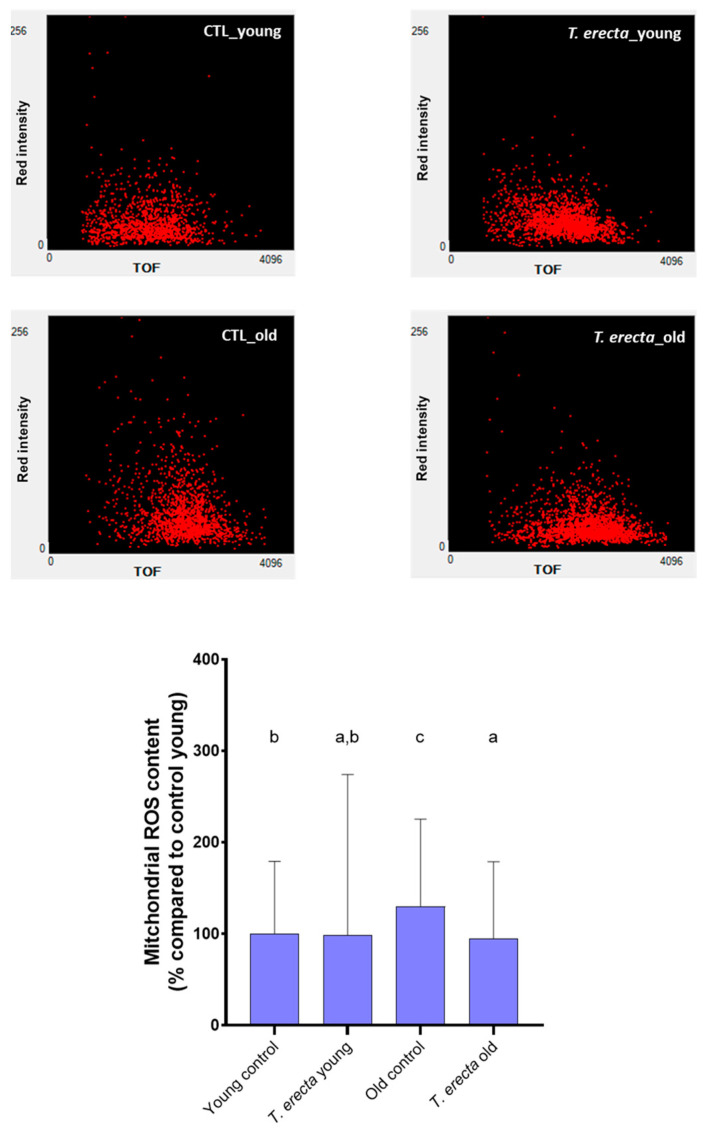
Mitochondrial ROS content in Young and Old worms. Mitochondrial ROS content was measured by the Mitotracker Red CM-H2 XRos assay. L3 stage synchronized N2 nematodes were treated or untreated with 100 µg/mL *T. erecta* extract and incubated at 20 °C until the read-out point of the experiment (Young worms: 5 days old; Old worms: 12 days old). Representative images of Mitochondrial ROS quantification by Multi-Range Large Particle Flow Cytometer Biosorter (Union Biometrica, MA, USA) are shown above the bar charts (red fluorescence intensity vs. TOF: time of flight; worm size). Data are expressed as mean ± SD of three independent experiments (n = 3). Lower-case letters, when different, indicate statistically significant differences (*p* < 0.05). CTL_young: Young control (untreated worms); *T. erecta*_young: *T. erecta* treated worms (Young); CTL_old: Old control (untreated worms); *T. erecta*_old: *T. erecta* treated worms (Old).

Mitochondria are the main source of oxidative stress during aging. These cellular organelles are responsible for energy generation, as well as the regulation of cellular metabolism and oxidative stress [54]. Subsequently, mitochondrial ROS production was also evaluated (Figure 5). In this case, significant differences were only observed concerning treatment with the petal extract for the older worms. Treated older worms released less ROS in mitochondria than untreated ones. Mitochondria are particularly susceptible to ROS production due to their proximity to the generation of free radicals [55]. Therefore, the reduction in ROS may improve mitochondrial integrity and preserve cellular function.

**Figure 6:**
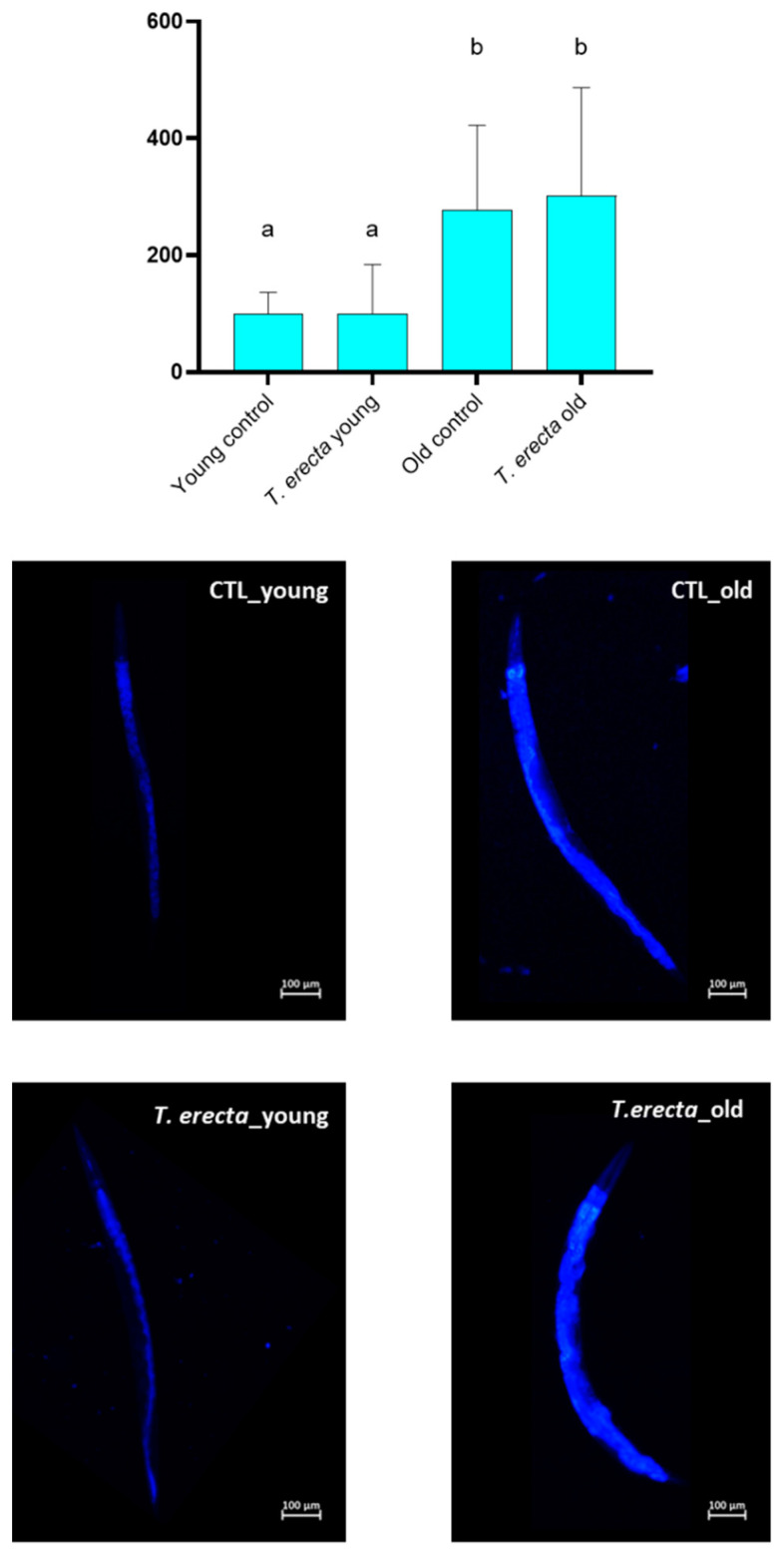
Lipofuscin content. Lipofuscin content was quantified from images acquired with a 10× objective lens and analyzed using NIS-Elements BR software (Nikon, Tokyo, Japan). L3 stage synchronized N2 nematodes were treated or untreated with 100 µg/mL *T. erecta* extract and incubated at 20 °C until the read-out point of the experiment (Young worms: 5 days old; Old worms: 12 days old). Representative images (10× magnification) of lipofuscin quantification by Nikon epi-fluorescence microscope (Eclipse Ni, Nikon, Tokyo, Japan) fitted with a Nikon DS-Ri2 camera (Tokyo, Japan) are shown below the bar charts. Data are expressed as mean ± SD of three independent experiments (n = 3). Lower-case letters, when different, indicate statistically significant differences (*p* < 0.05). CTL_Young: Young control (untreated worms); *T. erecta*_young: *T. erecta* treated worms (Young); CTL_old: Old control (untreated worms); *T. erecta*_old: *T. erecta* treated worms (Old).

Finally, the content of lipofuscin, a compound whose accumulation in tissues is associated with oxidative damage, and a marker of aging, was evaluated. In this case, no significant differences were found between BR5706 worms treated with the petal extract and untreated ones (Figure 6). Lipofuscin is a measure used as an indicator of oxidative damage in tissues and is considered to be a marker of cellular aging as it reflects the cells’ inability to efficiently eliminate oxidation products.

## 3. Materials and Methods

### 3.1. Edible Flower

The petals of *T. erecta* were kindly provided by the company Innoflower in Zaragoza, Spain (http://www.innoflower.com, accessed on 20 October 2023). These petals were cultivated at the facilities of the company situated in Zaragoza, Spain, and were picked during the months from April to September. These petals are commercially available and the catalog reference is TAGETE (Innoflower, Zaragoza, Spain) The samples were stored at −80 °C until the analysis was carried out.

### 3.2. Extract Obtention Procedure

The extract was obtained following the procedure described by L. Rivas-García et al. [22]. Briefly, *T. erecta* petals were dried by lyophilization using a vacuum pump (Telstar, Madrid, Spain). Then, 1 mL of the extraction solution, composed of ethanol/Milli-Q water/concentrated formic acid (80:20:0.1, *v*/*v*/*v*) was added to 500 mg of petals. Subsequently, they were homogenized by an Ultraturrax T25 homogenizer (Janke & Kunkel, IKA Labortechnik, Staufen, Germany) at medium–high speed for 2 min. The extraction efficiency was performed by agitating the suspension at 22 (ARE Magnetic stirrer, VELP Scientifica, Usmate, Italy) for 2 h in the absence of light at room temperature (RT). Then, two centrifugations at 2400× *g* for 15 min were carried out to separate solid particles. The supernatants were filtered using a 0.45-µm Minisart filter (PBI International, Milan, Italy) and transferred to a 5.0 mL amber glass container. Finally, the ethanolic extract was dried and concentrated using a rotary evaporator. The final sample was stored at −80 °C until required.

### 3.3. Extract Characterization

#### 3.3.1. Total Phenolic Content

To determine the total phenolic content (TPC), we included 100 mL of the Mexican marigold water-resuspended extract in 500 μL of Folin-Ciocalteu solution and maintained it at 4 °C in the absence of light. Following this, the mixture was allowed to incubate for 1 to 8 min at room temperature, and then 400 μL of 0.7 M sodium carbonate (Na_2_CO_3_) was incorporated. The solution was then left to incubate for 2 h at RT (approximately 23 °C) in the absence of light, and absorbance was measured at 760 nm using a Synergy Neo2 microplate reader (Biotek, Winooski, VT, USA). Gallic acid solutions ranging from 0.5 to 3.0 mM were utilized as the standard. The experiment was performed in triplicate.

#### 3.3.2. Total Flavonoid Content

The total flavonoid content (TFC) was measured using a colorimetric method. To achieve this, 250 μL of water-resuspended petal extract was combined with 1.25 mL of Milli-Q water, and a solution formed of 75 μL of a 5% sodium nitrate (NaNO_2_) was added. After a 6-min incubation, 150 μL of a 10% solution of aluminum chloride hexahydrate (AlCl_3_·6H_2_O) was added to the solution and left to mix for 5 min. Subsequently, 500 μL of 1 M sodium hydroxide (NaOH) was added, bringing the total volume to 2.5 mL with Milli-Q water, and absorbance was promptly measured at 510 nm using a Synergy Neo2 microplate reader (Biotek, Winooski, VT, USA). Solutions of (+)-catechin within the concentration range of 0.0125 to 0.1 mg/mL served as the standard. The experiment was performed in triplicate.

#### 3.3.3. Total Antioxidant Capacity

The total antioxidant capacity (TAC) of the petal’s fractions was determined using three different methodologies: the Ferric Reduced Antioxidant Process (FRAP) determination, the DPPH (2,2-diphenyl-1-picrylhydrazyl) free radical methodology, and the Trolox Equivalent Antioxidant Capacity (TEAC) test. The methodologies used have been described previously [56]. Briefly, for DPPH determination, 3 mL of the DPPH working solution (formed by 24 mg of DPPH in 100 mL of methanol) was mixed with 100 µL of extract. As a standard, a solution of 3 mL containing DPPH in 100 µL of methanol was also prepared. Subsequently, the tubes were placed in complete darkness for 15 min, and the absorbance was measured at 515 nm. For ABTS measurement, 10 μL of the extract was mixed with 990 μL of ABTS solution. Then, the solution was incubated at RT for 6 min in the absence of light, and the absorbance was measured at 734 nm.

Finally, for FRAP determination, 2.3 mL of the FRAP reagent was mixed with 0.7 mL of the extract at different concentrations (0.5–5.0 mg/mL). The mixture was then incubated at 37 °C for 30 min in the dark. The absorbance was determined at 593 nm.

### 3.4. HPLC-QTOF-MS/MS

All the chemicals used for this assay were LC-MS grade and the water used was Milli-Q type obtained from Millipore purification equipment. The acetonitrile, methanol, ethanol, and formic acid used were purchased from Fisher Scientific (Hampton, VA, USA) and Fluka (Sigma-Aldrich, St. Louis, MO, USA). The analytical standards for quantification were p-coumaric, gallic acid, luteolin, luteolin 7-glucoside, quercetin, quercetin-3-glucoside, quinic acid, sucrose, and syringic acid. All standards were provided by Sigma-Aldrich (St. Louis, MO, USA). The main analytical parameters of the quantification method are detailed in Supplementary Table S1. Mass spectrometry reference solution for ESI from Agilent Technologies (Santa Clara, CA, USA) was used for daily mass calibration. The sample was analyzed using the methodologies previously described in the bibliography [25,28,57]. Briefly, the extract was analyzed in triplicate using an Agilent 1260 series liquid chromatograph equipped with a micro-vacuum degasser, binary pump, thermostatic autosampler and column compartment, and diode array detector. To separate the components of the sample, an Agilent Zorbax Eclipse Plus C18 column with dimensions of 4.6 × 150 mm and a particle size of 1.8 μm was utilized. The mobile phases used were water with 0.1% formic acid as phase A and acetonitrile as phase B, with the following gradient: 0 min, 5% phase B; 20 min, 50% phase B; 28 min, 95% phase B; 29 min, 5% phase B, and finally, a 7 min conditioning cycle with the initial analysis conditions to equilibrate the system. The flow rate used was 0.5 mL/min, the column temperature was maintained at 25 °C, and the autosampler was maintained at 4 °C. The injection volume was 5 μL. Compound detection was performed with an Agilent 6540 Ultra High Definition (UHD) Accurate Mass Q-TOF detector equipped with a dual ESI Jet Stream interface. Detection by QTOF was performed in negative ionization mode in a mass range of 50–1700 *m*/*z*. Ultrapure N2 was used as the ionization and drying gas at temperatures of 325 °C and 400 °C, respectively, and flows of 10 and 12 L/min, respectively. Other parameters were as follows: capillary voltage, 4000 V; N2 pressure in nebulizer, 20 psig; Q1 voltage, 130 V; nozzle voltage, 500 V; skimmer, 45 V; octopole 1 RF, 750 V. The analysis was performed with continuous ionization of trifluoroacetate anion (112.985587 *m*/*z*) and a hexakis (1H,1H,3H-tetrafluoropropoxy) phosphazine adduct (1033.988109 *m*/*z*) with the aim of recalibrating each mass spectrum acquired during the analysis. Additionally, MS/MS analyses were performed in automatic fragmentation mode, isolating and fragmenting the two most intense mass peaks, with the following collision energy values: 10 eV, 20 eV, and 40 eV. MS/MS data were acquired using the centroid mode at a rate of 2.5 spectra/s in the extended dynamic range mode (2 GHz). All data acquisition operations were controlled with Masshunter workstation software version B.06.00 (Agilent Technologies, Santa Clara, CA, USA). The main compounds in the extract were automatically detected using a compound extraction algorithm based on molecular feature detection, and the resulting peaks were filtered with a relative volume threshold of 0.2% in addition to those that appeared in the solvent blank. Whenever possible, the compounds detected by this algorithm were tentatively identified with the help of compound databases (Scifinder, HMDB, Metlin, etc.) and scientific literature related to *T. erecta* [25,28,57], based on the molecular formula obtained from the exact mass and isotopic distribution data, retention times, and fragmentation patterns recorded. The identified compounds were quantified based on MS detection and using surrogate standards while the Extracted Ion Chromatogram (EIC) of each individual metabolite was employed. The surrogate analytical standards used are listed in Supplementary Table S2.

### 3.5. In Vitro Assays

#### 3.5.1. Cell Conditions

A2780 cells were provided by the CIC at the University of Granada. Ovarian cells were maintained in the Roswell Park Memorial Institute RPMI-1640 Medium (Sigma-Aldrich, St. Louis, MO, USA) with a 10% (*v*/*v*) supplementation of fetal bovine serum (FBS) (Sigma-Aldrich, St. Louis, MO, USA) and 2 mM of L-glutamine (Sigma-Aldrich, St. Louis, MO, USA). The flasks that contained the cells were incubated in controlled conditions of 37 °C in a humidified atmosphere of 5% CO_2_ and the medium was changed every 2–3 days. Cells were detached when they became confluent by using trypsin-EDTA solution (Sigma-Aldrich, St Louis, MO, USA).

#### 3.5.2. Cell Viability Test

Cell viability was assessed through the MTS assay, which relies on the conversion of a tetrazolium salt, specifically 3-(4,5-dimethylthiazol-2-yl)-5-(3-carboxymethoxyphenyl)-2-(4-sulfophenyl)-2H-tetrazolium (MTS), into formazan by metabolically active cells. To perform this assay, 1 × 10^5^ A2780 cells per mL were seeded in 96-well plates (VWR, Radnor, PA, USA) and cultured for 24 h. Subsequently, the culture medium was replaced and various concentrations of *T. erecta* extract (10 μg/mL, 50 μg/mL, 100 μg/mL, 250 μg/mL, 500 μg/mL, and 1000 μg/mL, dissolved in MilliQ water as a vehicle control) were added. After 48 h of incubation, cell viability was measured using the CellTiter 96 AQueous non-radioactive cell proliferation assay, following the manufacturer’s guidelines (Promega, Madison, WI, USA). Absorbance at 490 nm was determined by a microplate reader (Biotek, Winooski, VT, USA). Finally, the IC_50_ concentration was calculated from viability-concentration curves using GraphPad Prism 8 software.

#### 3.5.3. Cell Death Mechanism: Autophagy Induction

Autophagy induction was measured using the CYTO-ID ENZKIT Autophagy Detection Kit 2.0 (Enzo Life Sciences, Lausen, Switzerland) following the instructions provided by the manufacturer. The quantification of the autophagy induction was performed by flow cytometry using Becton Dickinson Flow Cytometer (Franklin Lakes, NJ, USA) equipped with the FACS Diva 6.1 software program (BD Biosciences, San Jose, CA, USA). For the analysis, we acquired at least 1 × 10^5^ events/sample. The data is presented as the mean of three independent experiments.

#### 3.5.4. Cell Death Mechanism: Apoptosis and Necrosis

A dual staining procedure utilizing annexin V/fluorescein isothiocyanate (FITC) and propidium iodide (PI) was used to assess apoptosis and necrosis production in A2780 cells. Cells were plated in 35 mm culture dishes (VWR, PA, USA) at a density of 2 × 10^5^ cells/mL and cultured under the conditions described in Section 3.3.1. After 24 h, the culture medium was removed and replaced with 1 mL of fresh medium containing either the IC_50_ of *T. erecta* extract or 0.2 nM of cisplatinum (used as a positive control). Subsequently, the cells were incubated for 48 h, harvested through trypsinization, and then centrifuged at 1000× *g* for 5 min at RT. The resulting pellet was washed twice with 1 mL of cold Phosphate Buffered Saline (PBS) 1×, followed by centrifugation at 1000× *g* for 5 min after each wash. Following these centrifugation steps, 100 μL of annexin binding buffer 1×, 5 μL of annexin V-FITC, and 2 μL of PI (Annexin V-FITC Apoptosis Detection Kit, Invitrogen, Waltham, MA, USA) were added to all the samples and incubated for 15 min at room temperature in the absence of light. Subsequently, 400 μL of annexin binding buffer 1× and 500 μL of cold PBS 1× were added to these cell suspensions. The quantification of apoptotic events and necrosis was developed by flow cytometry on a Becton Dickinson Flow Cytometer (Franklin Lakes, NJ, USA) using the FACS Diva 6.1 software program (BD Biosciences, San Jose, CA, USA), with the acquisition of at least 1 × 10^5^ events/sample. Experiments were performed in triplicate.

#### 3.5.5. ROS Production

ROS production was determined by employing 2′-7′ dichlorofluorescin diacetate (DCFH-DA), a cell-permeable fluorogenic dye reagent that detects the presence of hydroxyl, peroxyl, and other ROS activities within the cells. Once taken up by the cells, DCFH-DA is deacetylated by cellular esterases, transforming it into a non-fluorescent compound. Subsequently, ROS oxidizes it into 2′-7′ dichlorofluorescein (DCF). To measure ROS generation, flow cytometry was performed using a Becton Dickinson Flow Cytometer located in New Jersey, USA, and equipped with FACS Diva 6.1 software from BD Biosciences in California, USA. A dose of 25 μM of hydrogen peroxide (H_2_O_2_) was used as a positive control.

### 3.6. Caenorhabditis Elegans Assays

#### 3.6.1. Maintenance of *C. elegans*

*C. elegans* were obtained from the CGC (Minneapolis, MI, USA) and housed at 20 °C on solid NGM plates fed with Escherichia coli OP50 in an incubator (VELP Scientifica FOC 120 E, Usmate, Italy). The strain used for all the experiments was BR5706 (bkIs10). Additionally, for the lethality test N2 Bristol (wild type) worms were employed. For the experiments, a bleaching method was used to obtain age-matched embryos according to standard protocols [58]. Briefly, worms were washed and collected with M9 buffer and embryos were isolated using a bleaching solution (sodium hypochlorite 4% and NaOH 0.5 N [20/80; *v*/*v*]). The embryos were then washed three times and dispensed into the experimental plates.

#### 3.6.2. Tau Proteotoxicity Assessment

The effect of *T. erecta* petal extract treatment on locomotive parameters in a *C. elegans* model of tauopathy was analyzed with the objective of evaluating an extensive field of the pathophysiology of AD. The BR5706 strain shows a constitutive pan-neuronal expression of pro-aggregant human tau protein which results in the deposition of aggregates leading to locomotion defects noted mainly from day one of adulthood. BR5706 worms were incubated at 20 °C from eggs on NGM containing either the treatment or untreated for 72 h. Then, these animals were forced to swim to stimulate worm movement. For this purpose, at least 20 worms were transferred to a slide with a drop of M9 and worm movement was recorded, tracked, and analyzed with the WormLab Imaging System (MBF Bioscience, Williston, VT, USA). Swimming speed, activity index, and wavelength were evaluated as representative parameters of locomotive behavior.

#### 3.6.3. Intracellular ROS Content Determination

Intracellular ROS content was measured by the 2′,7′-dichlorofluorescein diacetate (H2DCFDA) assay. L3 stage synchronized N2 nematodes were treated or untreated with 100 µg/mL of Mexican marigold petal extract and incubated at 20 °C until the read-out point of the experiment (Young worms: 5 days old; Old worms: 12 days old). In the old population, 15 μg/mL FUDR (Sigma-Aldrich, St. Louis, MO, USA) was applied in order to avoid egg laying during the fertile phase. Furthermore, the animals were moved to new plates with fresh treatment and food twice per week in the case of the aged worms, and once per week for the young group. After that time, all nematodes were washed with M9 medium and incubated for 2 h with 25 μM DCFDA at 20 °C. The fluorescence intensity was determined by a Multi-Range Large Particle Flow Cytometer Biosorter (Union Biometrica, Holliston, MA, USA). At least 300 worms were used per group. The results are expressed as a percentage of the young control using the mean of the yellow fluorescence intensity, which is related to the ROS content.

#### 3.6.4. Mitochondrial ROS Content Determination

Mitochondrial ROS content was measured by the Mitotracker Red CM-H2 XRos assay (Thermo Fisher, Waltham, MA, USA). L3 stage synchronized N2 nematodes were treated or untreated with 100 µg/mL of *T. erecta* extract and incubated at 20 °C until the read-out point of the experiment (Young worms: 5 days old; Old worms: 12 days old). In the old population, 15 μg/mL FUDR (Sigma-Aldrich, St. Louis, MO, USA) was applied in order to avoid egg laying during the fertile phase. Furthermore, the animals were moved to new plates with fresh treatment and food twice per week in the case of the aged worms, and once per week for the young group. After that time, all nematodes were washed with M9 medium and transferred to NGM plates containing 10 μM Mitotracker mixed with dead *E. coli* OP50 as a food source. Worms were incubated with the dye for 2 h at 20 °C and then fluorescence intensity was measured using a Multi-Range Large Particle Flow Cytometer Biosorter (Union Biometrica, Holliston, MA, USA). At least 300 worms were used per group. The results are expressed as a percentage of the young control using the mean of the red fluorescence intensity, which is related to the mitochondrial ROS content.

#### 3.6.5. Lipofuscin Content Quantification

L3 stage synchronized N2 nematodes were treated or untreated with 100 µg/mL of *T. erecta* extract and incubated at 20 °C until the read-out point of the experiment (Young worms: 5 days old; Old worms: 12 days old). In the old population, 15 μg/mL FUDR (Sigma-Aldrich, St. Louis, MO, USA) was applied in order to avoid egg laying during the fertile phase. Furthermore, the animals were moved to new plates with fresh treatment and food twice per week in the case of the aged worms, and once per week for the young group. After that time, the worms were mounted on glass slides with M9 medium and sodium azide. Fluorescence images were captured with a Nikon epi-fluorescence microscope (Eclipse Ni, Nikon, Tokyo, Japan) fitted with a Nikon DS-Ri2 camera (Tokyo, Japan). The images, acquired with a 10× objective lens, were analyzed using NIS-Elements BR software, v.5.2 (Nikon, Tokyo, Japan). Autofluorescent worms were viewed with the blue filter (DAPI). A minimum of 30 worms per group were measured and results were expressed as a percentage of the young control using the average of the total fluorescence intensity for each worm.

#### 3.6.6. Acute Lethal Toxicity Assay

Acute toxicity assay was carried out using the wild-type N2 synchronized L4 following the methodology proposed by Navarro-Hortal and collaborators [50]. The results obtained from this assay were employed to select the concentration for the assays.

### 3.7. Figure Generation

Parts of the figure were drawn by using pictures from Servier Medical Art. Servier Medical Art by Servier is licensed under Creative Commons Attribution 3.0 and Pixabay.

### 3.8. Statistical Analysis

The normality of the data was assessed using the Kolmogorov–Smirnov test, and variance homogeneity was examined using the Levene test for all variables. Different statistical tests were applied depending on the distribution of the variables: the Student’s *t*-test for normally distributed variables and the Mann–Whitney U and Kruskal–Wallis tests for non-normally distributed variables. The results were based on a minimum of three independent experiments and are presented as the mean ± standard error of the mean for all experiments. Statistical significance was considered for *p*-values less than 0.05. Statistical analysis was performed using SPSS 24.0 (IBM, Armonk, NY, USA) and GraphPad Prism 9 software (GraphPad Software, Boston, MA, USA) was utilized to generate graphical representations.

## 4. Conclusions

The present study has demonstrated the significant antioxidant activity of the extract derived from *T. erecta* petals. This antioxidant effect can be attributed to the high polyphenolic content of the extract, wherein patulitrin, methoxyquercetin, and quercetagetin were particularly abundant. In addition, a total of 36 different metabolites were identified in the extract studied. The results revealed promising preliminary activity of this Mexican marigold extract in mitigating age-related non-transmissible diseases including AD and cancer. Treating ovarian tumor cells with the extract obtained from *T. erecta* showed a dose-response effect; moreover, treatment with the extract induced apoptosis activation as a mechanism of cell death. On the other hand, the extract reported promising findings against AD. Thus, in a *C. elegans* tauopathy model, the *T. erecta* extract has the ability to reduce the production of ROS. However, these promising effects need to be corroborated in other experimental models such as murine models.

These findings suggest that the Mexican marigold petal extract may hold promise as a potential therapeutic agent, particularly in conditions where oxidative stress plays a significant role, such as the aging process. This study underscores the potential of *T. erecta* and could be considered for its application in several steps for functional food development.

## Figures and Tables

**Table 1:** Total phenolic and flavonoid content and total antioxidant capacity of the Mexican marigold ethanolic extract.

Parameter	Mean ± SEM
Total phenolic content (mg GAE/g extr)	81.1 ± 10.6
Total flavonoid content (mg catechin/g extr)	23.17 ± 4.26
FRAP (μM trolox/g extr)	1475.3 ± 122.5
DPPH (μM trolox/g extr)	1950.3 ± 211.1
ABTS (μM trolox/g extr)	977.7 ± 62.4

ABTS: 2,2′-azino-bis(3-ethylbenzothiazoline-6-sulfonic acid) method. DPPH: 2,2-diphenyl-1-picrylhydrazyl free radical method. extr: extract. GAE: Gallic acid equivalent. FRAP: ferric-reducing antioxidant power assay.

**Table 2:** Compounds identified and quantified in *T. erecta* extract.

Formula	RT (min)	[M-H]^−^, *m*/*z*	Mass	Proposed Compound	Concentration (mg Standard eq/g Extract ± SD)	Peak in Figure 1
C_7_H_12_O_6_	3.42	191.0569	192.0641	Quinic acid ^a^	5.49 ± 0.02	1
C_13_H_22_O_11_	3.55	707.2265	354.1168	Quinic acid hexoside isomer 1 ^a^	0.20 ± 0.01	2
C_13_H_22_ O_11_	4.9	707.2267	354.1176	Quinic acid hexoside isomer 2 ^a^	0.16 ± 0.01	3
C_14_H_16_O_10_	7.47	343.0676	344.0749	Theogallin isomer 1 ^b^	0.3 ± 0.002	4
C_14_H_16_O_10_	9.06	687.1427	344.0749	Theogallin isomer 2 ^b^	0.22 ± 0.01	5
C_12_H_22_O_9_	9.94	309.1201	310.1273	Deoxy-fructofuranosyl deoxy-glucopyranoside/fructofuranosyl dideoxy-xylo-hexopyranoside ^c^	76.0 ± 3.00	6
C_26_H_30_O_19_	11.69	645.1322	646.1393	Digalloyl-dihexoside ^b^	0.51 ± 0.01	7
C_21_H_20_O_14_	11.95	495.0793	496.0864	Digalloylquinic acid ^b^	0.29 ± 0.003	8
C_20_H_20_O_14_	12.09	483.0785	484.0857	Digalloyl-hexoside ^b^	0.08 ± 0.001	9
C_19_H_26_O_12_	12.12	445.1359	446.1428	[(Xylopyranosyl-glucopyranosyl)oxy]benzeneacetic acid ^c^	0.92 ± 0.01	10
C_33_H_34_O_23_	12.2	797.1436	798.1506	Trigalloyl-dihexoside ^b^	0.53 ± 0.01	11
C_28_H_32_O_18_	12.31	655.1525	656.1596	Patuletin gentiobioside ^d^	0.63 ± 0.02	12
C_16_H_18_O_9_	12.9	353.0884	354.0956	Caffeoylquinic acid ^a^	0.26 ± 0.004	13
C_24_H_28_O_15_	13.15	555.1363	556.1435	Syringic acid-(dihydroxydimethoxybenzoic acid)-hexoside isomer 1 ^e^	1.8 ± 0.1	14
C_24_H_28_O_15_	13.39	555.1366	556.1438	Syringic acid-(dihydroxydimethoxybenzoic acid)-hexoside isomer 2 ^e^	4.0 ± 0.1	15
1C_20_H_28_O_12_	13.66	459.1514	460.1586	Apiopaeonoside/paeonolide ^c^	1.35 ± 0.04	16
C_20_H_16_O_13_	13.77	463.0522	464.0594	Ellagic acid-hexoside ^b^	0.07 ± 0.002	17
C_15_H_14_O_10_	14.59	353.0524	354.0596	Coumaroylhydroxycitric acid ^f^	2.5 ± 0.1	18
C_28_H_32_O_18_	14.72	655.1519	656.1591	Patuletin 3-gentiobioside ^d^	0.55 ± 0.01	19
C_21_H_20_O_13_	14.88	959.1753	480.0919	Quercetagetin-3-O-hexoside ^d^	4.0 ± 0.04	20
C_28_H_24_O_17_	15.13	631.0946	632.1017	Quercetagetin-7-O-(galloyl-hexoside) ^d^	0.5 ± 0.01	21
C_24_H_28_O_14_	15.32	539.1412	540.1484	Di-syringic acid hexoside isomer 1 ^e^	2.25 ± 0.04	22
C_24_H_28_O_14_	15.66	539.1409	540.1481	Di-syringic acid hexoside isomer 2 ^e^	3.3 ± 0.1	23
C_22_H_22_O_13_	16.24	987.2069	494.1079	Patulitrin isomer 1 ^g^	76.0 ± 1.0	24
C_14_H_6_O_8_	16.36	300.9996	302.0068	Ellagic acid ^b^	0.12 ± 0.004	25
C_29_H_26_O_17_	16.45	645.1109	646.1180	Caffeoyl-digalloyl—glucopyranose ^b^	0.298 ± 0.005	26
C_16_H_16_O_10_	16.71	367.0682	368.0754	Methoxy-oxo-benzopyranyl glucopyranosiduronic acid ^c^	12.4 ± 0.3	27
C_29_H_26_O_16_	16.95	629.1151	630.1223	Coumaroyl-digalloylglucose/coumaroyl-digalloyl-glucopyranoside ^f^	0.336 ± 0.1	28
C_22_H_22_O_13_	17.45	493.0994	494.1066	Patulitrin isomer 2 ^g^	4.3 ± 0.1	29
C_24_H_22_O_14_	17.51	533.0941	534.1013	Luteolin (malonylglucoside)/apigenin (malonylglucoside)/kaempferol (malonyl-glucoside) ^h^	1.6 ± 0.1	30
C_22_H_22_O_12_	17.65	477.1048	478.1120	Isorhamnetin hexoside ^h^	22.6 ± 0.2	31
C_15_H_10_O_8_	18.08	317.0312	318.0384	Quercetagetin ^d^	22.0 ± 1.0	32
C_22_H_22_O_13_	18.1	493.0992	494.1064	Patulitrin isomer 3 ^g^	4.2 ± 0.1	33
C_15_H_10_O_6_	21.38	285.0415	286.0487	Luteolin ^i^	0.45 ± 0.02	34
C_16_H_12_O_8_	21.58	331.0469	332.0542	Methoxyquercetin ^j^	47.0 ± 1.0	35
C_16_H_12_O_7_	24.06	315.0519	316.0591	Isorhamnetin ^i^	0.98 ± 0.04	36

^a^ quantified based on quinic acid; ^b^ quantified based on gallic acid; ^c^ quantified based on sucrose; ^d^ quantified based on quercetin-3-glucoside; ^e^ quantified based on syringic acid; ^f^ quantified based on p-coumaric acid; ^g^ quantified based on luteolin-glucoside; ^h^ quantified based on luteolin 7-glucoside; ^i^ quantified based on luteolin; ^j^ quantified based on quercetin.

## Data Availability

The data presented in this study are available on request from the corresponding author. The data are not publicly available yet because funded grants are still ongoing.

## Supplementary Materials

The supporting information can be downloaded at https://www.mdpi.com/article/10.3390/ijms25010280/s1.
