# Supplementary material for: Bioactive Properties of Tagetes erecta Edible Flowers: Polyphenol and Antioxidant Characterization and Therapeutic Activity against Ovarian Tumoral Cells and Caenorhabditis elegans Tauopathy
Source: Int J Mol Sci. 2023 Dec 24;25(1):280. doi: 10.3390/ijms25010280 (PMC10778855; doi:10.3390/ijms25010280)
Supplement: Supplementary file 1 [file ijms-25-00280-s001.zip › ijms-2712866-supplementary.pdf]

Standard	Mass	Formula	Calibration range (µg/mL)	Calibration curve	R^2
p-Coumaric	164.0473	$C_9H_8O_3$	0.5 – 15	y = - 12488.1 x_2 + 596452.1 x – 8494.1	0.9906
Gallic acid	170.0215	$C_7H_6O_5$	0.5 – 15	y = - 36962.3 x_2 + 1883997.3 x + 114177.4	0.9985
Luteolin	286.047	$C_{15}H_{10}O_6$	0.5 – 15	y = - 53817.6 x_2 + 1784320.5 x + 1013628.1	0.9913
Luteolin 7-glucoside	448.1005	$C_{21}H_{20}O_{11}$	0.5 – 15	y = - 3145.9 x_2 + 105680.8 x + 535.3	0.9941
Quercetin	302.0426	$C_{15}H_{10}O_7$	0.5 – 15	y = - 58889.5 x_2 + 1899022.5 x + 561276.6	0.9910
Quercetin-3-glucoside	464.0954	$C_{21}H_{20}O_{12}$	0.5 – 15	y = - 13507.1 x_2 + 478124.4 x – 24299.9	0.9926
Quinic acid	192.0633	$C_7H_{12}O_6$	0.5 – 15	y = 31287.8 x_2 + 1208987.1 x – 261232.9	0.9936
Sucrose	342.1162	$C_{12}H_{22}O_{11}$	1 – 15	y = - 3382.0 x_2 + 174797.2 x + 49770.8	0.9972
Syringic acid	198.0528	$C_9H_{10}O_5$	0.5 – 15	y = - 2984.2 x_2 + 162372.9 x – 26661.2	0.9952

Supplementary Table S1. Main analytical parameters of the quantification method.

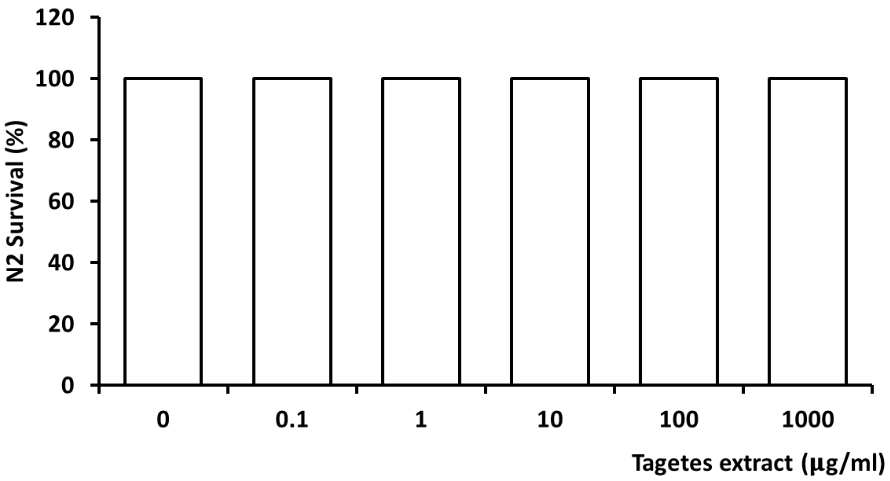

Supplementary Figure S1. Twenty-four hours lethality assay in the N2 wild strain exposed to exponential concentrations of the *T. erecta* extract.

Cpd	Formula	RT	m/z	Mass	Score (MFG)	Score (MFE)	Error (ppm)	Error (mDa)	Score (MFE)	Vol	Vol %	MSMS information		Proposed compound
1	$C_7 H_{12} O_6$	3.42	191.0569	192.0641	84.35	100	-3.73	-0.72	100	4001082	4.5	m/z	Abund %	Quinic acid
2	$C_{13} H_{22} O_{11}$	3.55	707.2265	354.1168	81.95	80	-1.78	-0.63	80	1936868	2.18	m/z	Abund %	Quinic acid hexoside isomer 1
3	$C_{13} H_{22} O_{11}$	4.9	707.2267	354.1176	95.55	100	-4.05	-1.43	100	1863902	2.1	m/z	Abund %	Quinic acid hexoside isomer 2
4	$C_{14} H_{16} O_{10}$	7.47	343.0676	344.0749	82.64	80	-1.47	-0.5	80	1002565	1.13	m/z	Abund %	Theogallin isomer 1
5	$C_{14} H_{16} O_{10}$	9.06	687.1427	344.075	82.72	100	-2	-0.69	100	2626790	2.96	m/z	Abund %	Theogallin isomer 2
6	$C_{12} H_{22} O_9$	9.94	309.1201	310.1273	96.55	100	-3.06	-0.95	100	4028228	4.53	–		Deoxy-fructofuranosyl deoxy-glucopyranoside / Fructofuranosyl dideoxy-xylo-hexopyranoside
7	$C_{26} H_{30} O_{19}$	11.69	645.1322	646.1393	96.05	100	-1.78	-1.15	100	1377822	1.55	–		Digalloyl-dihexoside
8	$C_{21} H_{20} O_{14}$	11.95	495.0793	496.0864	76.83	100	-2.26	-1.12	100	1080719	1.22	m/z	Abund %	Digalloylquinic acid
9	$C_{20} H_{20} O_{14}$	12.09	483.0785	484.0857	79.92	100	-0.72	-0.35	100	241502	0.27	m/z	Abund %	Digalloyl-hexoside
10	$C_{19} H_{26} O_{12}$	12.12	445.1359	446.1428	47.49	80	-0.82	-0.37	80	278992	0.31	m/z	Abund %	[(Xylopyranosyl-glucopyranosyl)oxy]benzeneacetic acid (synonymous Lucuminic acid)
11	$C_{33} H_{34} O_{23}$	12.2	797.1436	798.1506	94.88	80	-1.9	-1.52	80	1447647	1.63	m/z	Abund %	Trigalloyl-dihexoside
12	$C_{28} H_{32} O_{18}$	12.31	655.1525	656.1596	73.15	100	-1.17	-0.77	100	396625	0.45	m/z	Abund %	Patuletin gentiobioside
13	$C_{16} H_{18} O_9$	12.9	353.0884	354.0956	82.5	100	-1.56	-0.55	100	1068240	1.2	m/z	Abund %	Caffeoylquinic acid
14	$C_{24} H_{28} O_{15}$	13.15	555.1363	556.1435	76.63	100	-1.17	-0.65	100	497651	0.56	m/z	Abund %	Syringic acid-(dihydroxydimethoxybenzoic acid)-hexoside isomer 1
15	$C_{24} H_{28} O_{15}$	13.39	555.1366	556.1438	76.03	100	-1.67	-0.93	100	1341746	1.51	m/z	Abund %	Syringic acid-(dihydroxydimethoxybenzoic acid)-hexoside isomer 2
16	$C_{20} H_{28} O_{12}$	13.66	459.1514	460.1586	79.76	100	-1.09	-0.5	100	667254	0.75	m/z	Abund %	Apiopaeonoside / Paeonolide
17	$C_{20} H_{16} O_{13}$	13.77	463.0522	464.0594	80.33	95	-0.62	-0.29	95	410582	0.46	m/z	Abund %	Ellagic acid-hexoside
18	$C_{15} H_{14} O_{10}$	14.59	353.0524	354.0596	81.11	100	-2.52	-0.89	100	4298780	4.84	m/z	Abund %	Coumaroylhydroxycitric acid
19	$C_{28} H_{32} O_{18}$	14.72	655.1519	656.1591	73.9	100	-0.3	-0.2	100	275991	0.31	m/z	Abund %	Patuletin 3-gentiobioside
20	$C_{21} H_{20} O_{13}$	14.88	959.1753	480.0919	93.96	99.1	-3.08	-1.48	99.1	3107258	3.5	m/z	Abund %	Quercetagetin-3-O-hexoside
21	$C_{28} H_{24} O_{17}$	15.13	631.0946	632.1017	74.47	100	-0.6	-0.38	100	278003	0.31	m/z	Abund %	Quercetagetin-7-O-(galloyl-hexoside)
22	$C_{24} H_{28} O_{14}$	15.32	539.1412	540.1484	77.47	100	-0.85	-0.46	100	309447	0.35	m/z	Abund %	Di-syringic acid hexoside isomer 1
23	$C_{24} H_{28} O_{14}$	15.66	539.1409	540.1481	77.86	100	-0.39	-0.21	100	173248	0.21	–		Di-syringic acid hexoside isomer 2
24	$C_{22} H_{22} O_{13}$	16.24	987.2069	494.1079	92.9	100	-3.86	-1.91	100	6366134	7.16	m/z	Abund %	Patulitrin
25	$C_{14} H_6 O_8$	16.36	300.9996	302.0068	83.2	100	-1.81	-0.55	100	969996	1.09	m/z	Abund %	Ellagic acid
26	$C_{29} H_{26} O_{17}$	16.45	645.1109	646.118	96.93	100	-1.53	-0.99	100	930042	1.05	–		Caffeoyl-digalloyl--glucopyranose
27	$C_{16} H_{16} O_{10}$	16.71	367.0682	368.0754	96.4	100	-2.77	-1.02	100	3588600	4.04	m/z	Abund %	Methoxy-oxo-benzopyranyl glucopyranosiduronic acid
28	$C_{29} H_{26} O_{16}$	16.95	629.1151	630.1223	74.65	100	-0.39	-0.25	100	190455	0.21	m/z	Abund %	Coumaroyl-digalloylglucose / Coumaroyl-digalloyl-glucopyranoside
29	$C_{22} H_{22} O_{13}$	17.45	493.0994	494.1066	78.45	100	-1.17	-0.58	100	397745	0.45	m/z	Abund %	Patulitrin / Laricitrin-hexoside
30	$C_{24} H_{22} O_{14}$	17.51	533.0941	534.1013	77.96	100	-0.6	-0.32	100	333185	0.37	m/z	Abund %	Luteolin (malonylglucoside) / Apigenin (malonylglucoside) / Kaempferol (malonyl-glucoside)

31	$C_{22} H_{22} O_{12}$	17.65	477.1048	478.112	78.49	100	-1.78	-0.85	100	1067108	1.2	m/z	Abund %	Isorhamnetin hexoside
32	$C_{15} H_{10} O_{8}$	18.08	317.0312	318.0384	96.14	80	-2.51	-0.8	80	4577607	5.15	–		Quercetagetin
33	$C_{22} H_{22} O_{13}$	18.1	493.0992	494.1064	78.95	100	-0.67	-0.33	100	338850	0.38	m/z	Abund %	Patulitrin
34	$C_{15} H_{10} O_{6}$	21.38	285.0415	286.0487	81.43	80	-3.34	-0.96	80	2105097	2.37	m/z	Abund %	Luteolin
35	$C_{16} H_{12} O_{8}$	21.58	331.0469	332.0542	96.55	80	-3.05	-1.01	80	17033388	19.16	–		Methoxyquercetin
36	$C_{16} H_{12} O_{7}$	24.06	315.0519	316.0591	97.33	100	-2.54	-0.8	100	4732378	5.32	m/z	Abund %	Isorhamnetin

Supplementary Table S2. Additional information about the fragmentation parameters.
